# Positive Evolution of a Child Suffering from Caudal Regression Syndrome and Agenesia Sacra After Treatment with Growth Hormone and Rehabilitation

**DOI:** 10.3390/ijms26041627

**Published:** 2025-02-14

**Authors:** Jesús Devesa, Carla Fresco, Ana Devesa, Ana Rodríguez, Diego de Souza

**Affiliations:** 1 Scientific Direction, Medical Center Foltra, 15886 Teo, Spain; 2 Physiotherapy, Medical Center Foltra, 15886 Teo, Spain; carlafrescotouris@gmail.com; 3 BMRT, Medical Center Foltra, 15886 Teo, Spain; anadevesa81@gmail.com; 4 Hydrotherapy and Physiotherapy, Medical Center Foltra, 15886 Teo, Spain; anarodriguezalcover13@gmail.com; 5 Infirmary, Medical Center Foltra, 15886 Teo, Spain; desouzadiego8484@gmail.com

**Keywords:** caudal regression syndrome, gestational diabetes mellitus type I, GH, sacral agenesis, neurogenic bladder and bowel, Physiotherapy, Blomberg Rhythmic Movement Training, irreducible paraplegia

## Abstract

Caudal regression syndrome (CRS) is a malformation that occurs during the fetal period, and is mainly characterized by the incomplete development of the spinal cord (SC), which is often accompanied by other developmental abnormalities. The present study was performed in a 2-month-old boy with CRS, born to a type I diabetic mother, who presented interruption of the SC at the L5–L4 level, pelvic dislocation, sacral agenesis, hypoplastic femurs, lack of innervation of the lower limbs (spastic paraplegia), and a neurogenic bladder and bowel. Given the positive results we obtained in a previous study in a similar case, this patient was treated with GH (0.04 mg/kg/day, 5 days/week), melatonin (20 mg/day), and rehabilitation. The treatment only lasted 18 months, due to family problems. Blood tests and physical examinations were performed every 3 months initially and then every 6 months. Interestingly, despite GH administration, the child presented low plasma glucose and IGF-I values, which did not increase throughout the treatment, although there was significant growth of the patient, also indicated by elevated plasma alkaline phosphatase values. At the end of treatment, the gross motor function test (GMFM)-88 score increased from 0.93 (on admission) to 47.94. Sensory responses appeared in the lower limbs, and the patient was able to move his leg muscles in all directions and control his sphincters. Ten months after discharge, the patient was able to walk only with the aid of a back walker. GH treatment did not produce any adverse effects. In summary, despite the short duration of treatment, GH plus rehabilitation has been useful in innervating distal areas below the level of the incomplete spinal cord in CRS. GH likely acted on ependymal neural stem cells, as the hormone does on neurogenic niches in the brain, and rehabilitation helped achieve near-full functionality.

## 1. Introduction

Caudal regression syndrome (CRS) is a rare polymalformative syndrome that affects approximately 1–2 of every 100,000 live births [1,2,3], although its incidence increases in the presence of poorly controlled gestational diabetes, affecting up to 1 in every 300–350 newborns [4,5]. Although attempts have been made to explain the reasons for this difference in incidence, there are still no data that clarify it with certainty. Previous data suggested that CRS occurred predominantly in male newborns, but more recently it has been shown that the syndrome affects both sexes equally [6,7]. There have also been many divergences regarding the origin of this syndrome in relation to a possible genetic inheritance (for more details, see [3,8]). In fact, the syndrome that occurs in gestational diabetes may not differ, in terms of the many types of malformations, from that which occurs in normal pregnancies, and these may even be very diverse among affected patients. This does not exclude, however, that the cause of the syndrome, in all cases, is the result of the alteration of the expression of several genes in the early stages of fetal development. During fetal development, the expression of multiple genes occurs in a sequential and specific manner. It is therefore logical to think that the alteration in the expression of one or several genes during this period, especially in the first weeks of fetal life, the stage in which the vertebral column (SC) is formed, conditions the alteration in the expression of other genes that lead to abnormal development and the appearance of different pathologies, such as CRS.

In the case of CRS, this may be due to maternal or environmental toxic factors, including fetal dysregulation of retinoic acid homeostasis [9,10]. Maternal obesity, folate deficiency or the mother’s treatment with teratogenic medications during pregnancy increase the risk of developing a CRS [11]. Furthermore, maternal alcohol intake, poor oxygen supply to the fetus, and supposed amino acid imbalances have also been postulated as factors involved in the etiopathogenesis of CRS, but there is no clear evidence in this regard.

In any case, the main objective of this study is not to analyze the possible causes that lead to the development of CRS, a syndrome first described by Duhamel in 1962 [12], but to describe how it can be largely reversed, without further complications, after medical and rehabilitative treatment, something that we published for the first time in 2017 [12], when until then, and even today, it was considered that the only treatment for those suffering from CRS was amputation of the legs or orthopedic and supportive treatment [1,3].

The patient in this study was a 2-month-old boy with CRS, whose mother had been suffering from type I diabetes mellitus and on insulin treatment for 7 years. The spinal cord (SC) development of the patient had been interrupted at L4 (L5 was hypogenesic), and due to this, the patient presented so many neurological, organic, and bone abnormalities that his parents were told at his referral hospital that there were no viable solutions. Despite this, they came to our Medical Center, where we decided to treat him based on a similar case previously resolved satisfactorily [12], although its cause was likely genetic in origin. He had sacral and left renal agenesis, a neurogenic bladder and intestine, and a lack of sensory and motor innervation of the lower limbs. In addition, his pelvis was flexed, his legs were in irreducible spastic extension of 90° without any movement, there were hypoplastic femurs, and his feet were deformed. Although the parents had been told at their referral hospital that there were no viable solutions, we decided to treat him based on a similar case successfully resolved at our Medical Center [13]. The treatment was like that previously published by our group [13]: the daily administration of Growth Hormone (GH), melatonin, and physical rehabilitation. Although the treatment lasted only 18 months due to family problems, and of these 18 months, a total of about 4 months were interrupted by the parents’ vacations and 3 respiratory infections of the patient, the evolution upon discharge was very positive. The legs had grown and moved in all directions, the sensitivity in the legs was complete, the neurogenic bladder and intestine were no longer present, he was able to sit up and roll over on his own, and ten months after discharge, the patient was able to walk with the aid of a back walker and move independently on an electric motorcycle. The medical treatment did not produce any adverse side effects.

The importance of this study is that it is the second case in the world in which a solution has been found for a very severe pathology that until now, had no other solution than surgical and rehabilitation treatments merely to support the patient, while with our method, we have managed to resolve for the second time a large part of the developmental anomalies present in this type of patient, something that until now seemed impossible. In fact, we are already treating two other cases, although less severe—a boy and girl aged 4 and 5 years, respectively, who are already beginning to show significant positive changes. 

All of this opens up a new method of effective treatment for a majority of CRS patients. 

## 2. Results

### 2.1. Rehabilitation

**Figure 1:**
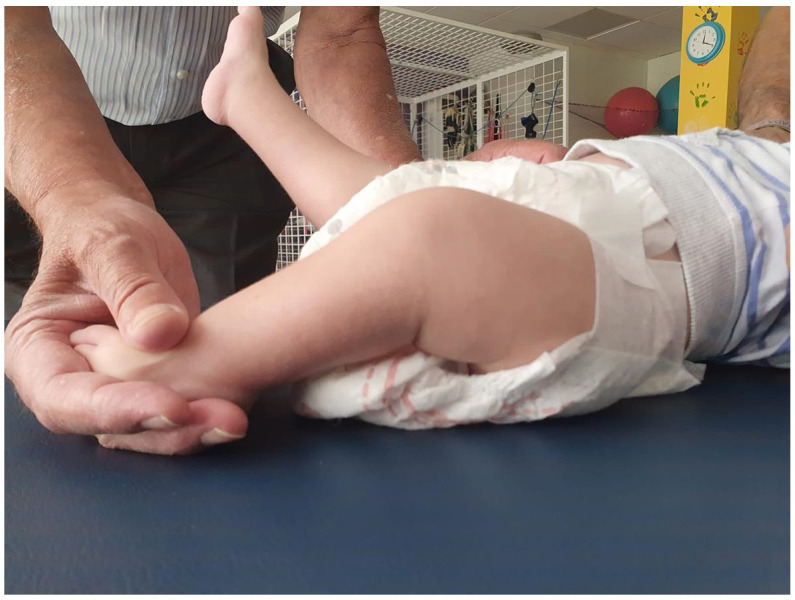
Induction of a flexion of approximately 25° in the left leg, something impossible at admission.

The first assessment in Physiotherapy, three months after starting treatment, indicated an improvement in the resting position, with a greater extension of the lower limbs close to horizontal (−30°, possibly due to hip flexion). There was also less resistance to passive extension. As for active mobility, the activation of both sides of the trunk increased, although the right side remains predominant. Dissociation appeared in the active movement of the lower limbs unilaterally, in addition to the initiation of active mobility in the extension of both legs of approximately 15°. At the knee level, a passive range of movement of approximately 5° on the right and 25° on the left was gained (Figure 1). 

Active flexion and extension of the right knee was also observed. The feet remained inactive, with no Babinski reflex, but with a starting position of the right foot closer to the midline and the left foot at a right angle of 90°. There was therefore a favorable evolution, despite the short time elapsed since the start of treatment, mainly regarding the active mobility of the lower limbs and the dissociation of both legs. 

Similarly, in BMRT, the therapist reported that during these first three months of treatment, a significant improvement had been observed in the patient compared to the situation on admission. Reflexes in the back, that did not exist before, began to appear, although in the lumbar area they were still more marked on the right side than on the left. The patient was now able to turn himself practically without help from prone to supine using his arms. There was greater mobility and muscle contraction detected in the thighs. Sensitivity appeared in the lower limbs, although there was still no response to the stimuli of the Babinski and plantar reflexes in either foot, although the patient seemed to notice the stimulus, since when the soles of his feet were touched, he reacted by moving his legs.

The patient’s adaptation to aquatic therapy was easy, and in the first months of treatment, he achieved the active mobility of both lower limbs, began pushing from flexion to extension, and acquired hand–leg coordination. He also gained ranges of motion of passive hip extension and the flexion of both knees.

**Figure 2:**
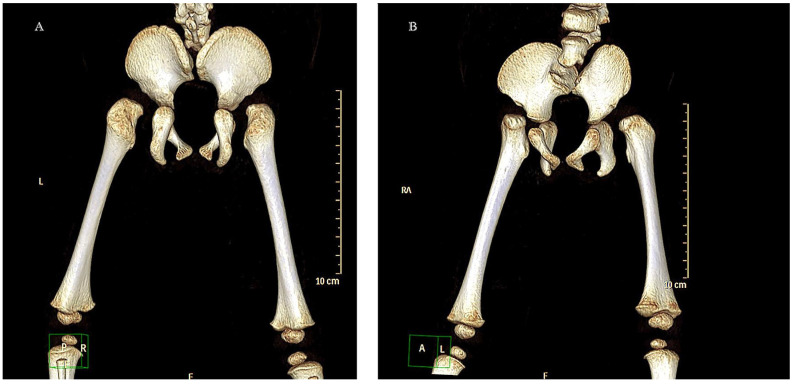
The images show complete agenesis of the Sacro coccyx and hypogenesic L5, leading to opposition of the iliac bones and consequent narrow pelvis. In Image B, the flexion of the pelvis and part of the spine can be seen. The study indicates the absence of the left kidney, as well as a partially filled bladder with thin walls. Note also the absence of pelvic acetabulum, the dislocation of the pelvis, but also that the femurs are increasing their growth. (**A**) L: Left; P: Posterior; R: Right; and F: Frontal. (**B**) RA: Right Anterior; A: Anterior; L: Left; and F: Frontal.

At 5 months of age, a helical CT scan of the lumbosacral structures was performed, with multiplanar and volumetric reconstructions (Figure 2).

One month later, an electroneurographic examination of the lower limbs was performed, and no motor responses were observed in the peroneal or tibial muscles (in both legs). At the sensory level, consistent and normal responses were observed in the left sural muscle. In the electromyographic examination of these lower limbs, hardly any insertion activity or voluntary activity was observed in the muscles explored below the knee, but there was in the thigh and gluteal muscles, where the tracings were normal. The report concluded that these findings were compatible with the distal involvement of the L5 and S1 myotomes, with an absence of voluntary activity below the knee.

During the episode of hospitalization for bronchiolitis, a chest X-ray showed a marked right diaphragmatic lobulation, which could indicate the presence of a congenital diaphragmatic hernia, something that has been associated with some cases of CRS. During the same admission, an ultrasound of the abdomen and pelvis showed complete normality, except for the absence of the left kidney. The right renal excretory tract was completely normal, without any dilation or vesicoureteral reflux. During this hospitalization, brain and spinal magnetic resonance imaging was also performed, which revealed a completely normal brain level and myelination appropriate for his age. The spinal cord showed a high distal level (at the level of D12), blunt in appearance, with a small hydro syringomyelia cavity in that area measuring 1 cm longitudinally, and without evident filum terminale. There was sacral agenesis with medialization of the iliac bones, and hypogenesis of L5, and to a lesser extent of L4. The thecal sac ended at the level of L2–L3, with a small amount of adjacent epidural fat. There was also a defect in the segmentation (partial fusion) of the body of C7 and D1.

**Figure 3:**
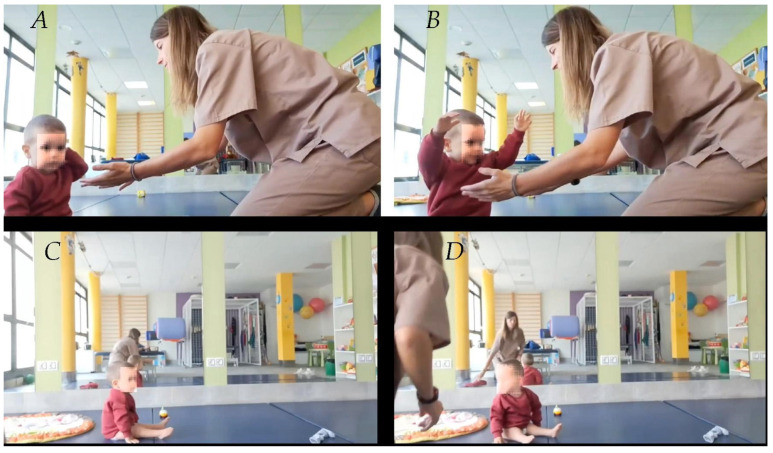
Age 14 months. The images show the following: (**A**) The patient is sitting with his legs extended on the floor. (**B**) He is trying to get the therapist to help him get up. (**C**) The patient is sitting normally waiting for the therapist. (**D**) The patient turns around on his own to greet the therapist. The face is disfigured to conceal his identity.

The last rehabilitation report, carried out three months (sixteen months of age) before the voluntary discharge of the patient, indicated that, compared to three months before, the patient was able to sit and remain with his legs extended and close to the ground, and even tried to get up with help (remember that at the beginning of the treatment the lower limbs were in a rigid position with a vertical extension of 90°) (Figure 3). 

**Figure 4:**
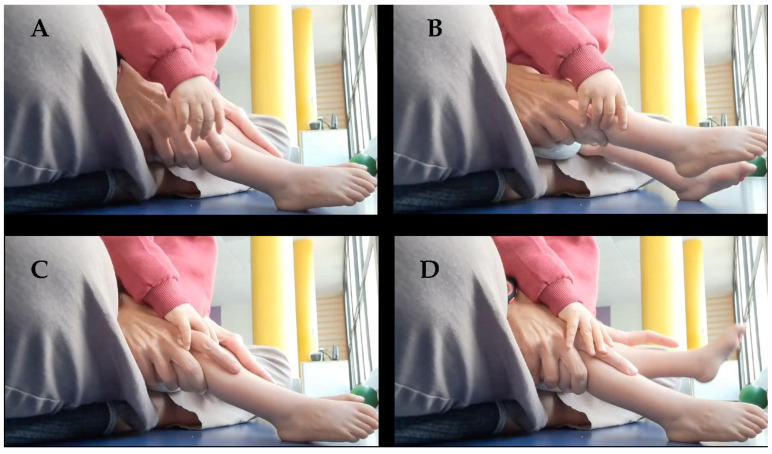
Age: 14 months. Unilateral voluntary movement of the legs. (**A**) The therapist’s index finger tells the patient that the right leg should be extended. (**B**) Voluntary extension of the right leg. (**C**) The therapist’s index finger tells the patient that the left leg only should be extended. (**D**) Voluntary extension of the left leg.

Regarding active mobility, he showed the ability to flex the lower limbs and return to a neutral position. Unilateral mobility of each lower limb was achieved, more effectively with the right leg (Figure 4), as well as active and voluntary movements of adduction and abduction of the legs.

At the knee level, flexion–extension was observed, more active in the right knee. The feet, however, remained without mobility, although the plantar reflex seemed to begin to appear. At the level of motor milestones, the patient had integrated an asymmetrical drag from the upper limbs and buttock walking when sitting, both backwards and forwards. He performed bipedalism with weight relief and adaptation in the position of the feet. There was rejection of active support of the feet. Sensitivity was full in the lower limbs, although the Babinski reflex was still absent. In summary, the patient continued to progress in the acquisition of motor milestones. 

**Figure 5:**
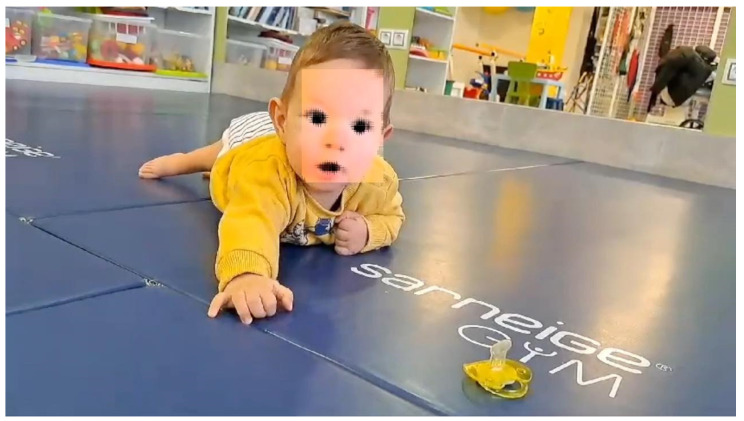
Age: 15 months. The patient may gate with the hands and arms trying to reach for an object on the floor. The face is disfigured to conceal his identity.

In relation to BMRT therapy, the last report indicated that the patient can now turn from face down to face up, the asymmetries between the right and left sides of the body have decreased, and that the Spinal Galant and Pérez reflexes have appeared on both sides of the back. The patient raised his head and crawled with his hands and arms (Figure 5), but there was still no reaction in the feet to different stimuli, apart from that mentioned above, although there was a response in the legs when these stimuli were employed.

The latest report on aquatic therapy focused on the patient’s total adaptation to the aquatic environment, active and dissociated mobility of the lower limbs, spontaneous mobility from flexion to neutral extension, coordination between hands and legs, and trunk straightening reactions in both sides of the body.

**Figure 6:**
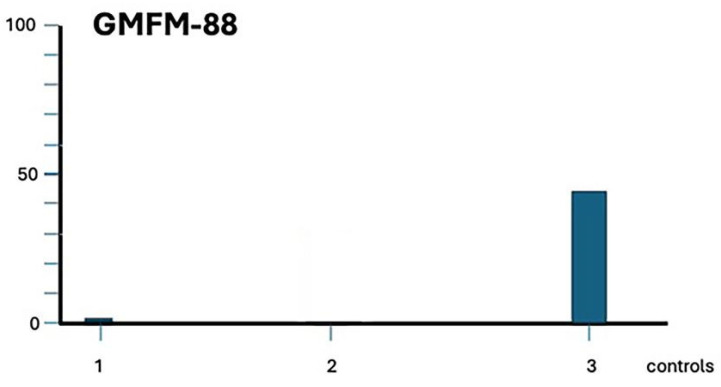
Graphical representation of the evolution of the Gross Motor Function test. The numbers on the *x*-axis indicate the time at which the assessments were carried out. 1: Evaluation after admission, at 2 months of age. 2: Evaluation scheduled to be carried out 9 months after the first evaluation, but it could not be carried out due to the patient’s absence. 3: Evaluation at 15 months of age. The patient was not yet able to walk on his own, partly because he had not fully recovered and partly because of his young age when that last assessment was conducted. Therefore, he had not yet reached level 100 on the scale. However, note the significant clear change in the scores achieved at 1 (0.93) and 3 (47.94).

Figure 6 shows the patient’s evolution according to the GMFM-88 scale.

### 2.2. Blood Analysis

At admission, blood tests were virtually normal, except for a plasma glucose value (62 mg/dL) slightly below the normal range (70–110 mg/dL). A low value of creatinine was also found (0.4 mg/dL, normal range: 0.5–1.4 mg/dL). Interestingly, plasma IGF-I values were very low for his age (27 ng/mL, normal range: 63–271 ng/mL, for pubertal stage Tanner I). Erythrocytes and Hb had normal values, as did the cells of the white series and the plasma proteins. Thyroid hormones and cortisol also had normal values. Despite the low IGF-I values and small size of the patient, we decided not to perform any provocative GH secretion test, partly because of his young age, but also because the child was going to be treated with this hormone. 

Subsequent tests were always normal, except for creatinine, which steadily remained at 0.4 mg/dL, and IGF-I, which peaked at 45 mg/dL (still below the normal range), although the patient was growing and alkaline phosphatase thus reflected this (415 U/L, normal range in children < 490 U/L). Blood sugar did not pass above 75 mg/dL at any time.

When the patient suffered the episode of bronchiolitis, the cells of the white series markedly increased, reaching total values of 15,000 leukocytes/mm^3^, of which 42.5% were neutrophils and 52.8% were lymphocytes, while erythrocyte, Hb, plasma protein and hormone levels remained within the normal range (except creatinine and IGF-I).

### 2.3. Medical Report 

It is important to note that despite the 18 months elapsed from admission to discharge from the Center, in a total of almost 4 months, spread over time, neither medical treatment nor rehabilitation therapies could be carried out, due to two episodes of respiratory infections and one of bronchiolitis suffered by the patient, and the vacations taken by the parents (Christmas, Easter, and summer). Despite this, we believe that the changes recorded in this severe condition are extremely important, starting with the disappearance of the neurogenic bladder and intestine. Although there was a continuous dribble of urine and feces upon admission and for several months, this gradually disappeared, and although due to his age he still did not voluntarily control his urinary and fecal emissions, these began to manifest abruptly, not continuously, as had previously occurred. Another important advance was that his hypoplastic and very small femurs upon admission grew by 12 cm by the time of his discharge, as well as the fact that the paralytic rigidity in the extension of his legs disappeared and he was able to move them to horizontal extension, independently flex them, and perform adduction and abduction movements.

The child did not present cognitive problems except for a delay in language development, and as stated before, brain structures and myelination were normal for his age. Blood tests were normal and there were no side effects attributable to the treatment, although it was striking that his plasma levels of IGF-I were always close to the lower limit of normal, despite treatment with GH and the child’s growth. 

## 3. Discussion

To our knowledge, this is the second case in the literature in which CRS is resolved favorably after treatment with GH, melatonin, and rehabilitation. This is very important, because until now, the treatment implied a multidisciplinary approach [13], including neurosurgery, orthopedic actuations for correcting deformities, and actuations addressed to maintain a normal renal function, as well as trying to control bladder and bowel incontinence. In fact, as stated in the Introduction, we recently began to treat two new cases of CRS in children aged 4 and 5 years old, much less affected than the two cases already resolved, since they only present an alteration of the gait and urinary and fecal incontinence. Prior to admission to our Center, both underwent Physiotherapy, without success, and suffered bladder catheterizations every 3 h. This is a clear expression that current supportive treatments depend on the severity of the syndrome and its multiple manifestations, but also that they are not aimed at correcting the syndrome as such. 

Of interest in this study is the fact that the patient was a product of maternal diabetes treated with insulin, while our previous study was carried out in a patient who was a product of a normal gestation [12]. This supposedly implies that the causes of the development of the syndrome were different, and yet the treatment worked equally well in both cases. Given this, the question that should be asked is why do two such different origins achieve such a similar result in their treatment. We should even ask ourselves why only a very small number of children of mothers with diabetes develop this syndrome.

To try to provide an answer to these questions, we will briefly analyze how embryonic development of the SC occurs in the early stages of fetal life, stages in which the typical malformations of CRS occur. 

Physiologically, somitogenesis is a critical process in embryogenesis. It begins at 20 days of embryonic age, during the neurulation process, when the somites sprout from the presomitic mesoderm (MSP), and follows a sequential and rhythmic organization along the anteroposterior axis of the embryo [14]. This rhythmic formation and progression of the somites seems to be dependent on the activation of specific signaling pathways involving many key genes and leading to a progressive wave of gene expression along the embryonic axis [12,14,15]. The result is the progressive and symmetrical bilateral formation of the somites during neurulation [14]. Once each somite is formed, the homeobox gene *Hox* is activated and determines the specificity of the somites along the anteroposterior axis of the embryo, although this only occurs when the newly formed somite has matured. It is then that the vertebrae and ribs are formed from the somites closest to the neural tube; the dorsal dermis is formed from the somites existing in the most dorsal region, while the skeletal muscles are formed from the cells present at the edges of each somite [14]. In addition, somites give rise to nerves, blood vessels, and connective tissue [16].

Logically, given the key role played at the physiological level by a series of genes in this process, any mutation or knockout, or alteration in the timing of expression, will cause damage of multiple types, depending on the genes affected or the age of development at which the affectation occurs in the normal development of the embryo. As we have already mentioned, these genetic alterations can be hereditary [3] or produced by toxic processes that affect the mother and consequently the fetus, but what are the reasons that the syndrome appears in the child of a diabetic mother, and why does it not occur in all children of mothers who suffer from this pathology?

Relatively recent estimates indicate that worldwide, 50% of women who become pregnant are obese or overweight [17], which implies a risk of suffering from gestational diabetes during pregnancy, something that, in fact, affects approximately 15% of pregnancies [18]. However, as we mentioned in the Introduction, CRS occurs in only 1 in every 300–350 pregnancies of a diabetic mother [4,5]. The explanation lies in what can happen during that short stage in the first weeks of gestation in which somitogenesis occurs, leading to the formation of the SC.

Maternal insulin does not cross the placenta, while the mother’s glucose does [19], and stimulates the secretion of insulin from the fetal pancreas, which develops at the 4th gestational week [20], although fetal insulin is not detected in plasma until the 12^th^ week of gestation [21]. This fetal insulin (and IGF-I) is responsible for fetal growth [22], but its excess leads to macrosomia [20], as well as an excess of fatty tissue [20], just what was observed (in relation to his gestational age) in the patient in our study after birth.

In the case of diabetic pregnant women, if glycemic control is poor, hyperglycemia will occur, which in turn will cause an increase in circulating glucose in the fetal plasma and the subsequent hypersecretion of insulin by the pancreas of that fetus. If the glycemic control of the diabetic pregnant woman is deficient in the first weeks of gestation when the process of somitogenesis takes place, and the fetal pancreas does not yet release insulin, fetal hyperglycemia could, theoretically, be the cause that leads to the development of CRS. If this were the case, the very different degrees of impact that the embryo may suffer would be related to the moment in which maternal glycemic control was deficient. Obviously, this is only a hypothesis that needs practical demonstration, but it seems to be the most logical response to the fact that only very few diabetic pregnant women have children with CRS. This hypothesis could also be the explanation for the case we are analyzing, since, although the mother had been on insulin treatment for 7 years, at the time of delivery, her Hb A1c was above the normal values for this parameter. Moreover, her body mass index (30.5, measured in our Center) indicated obesity.

In fact, this explanation has also been reported in a previous work [23], in which it was stated that deficient metabolic control of maternal glycemia at the time of conception and during the first months of pregnancy was critical during the differentiation of the precartilaginous mesenchyme, conditioning the fetus to skeletal malformations [24]. This must occur before day 28 of gestation because until that date is when the secondary neurulation completes the pending caudal segments that are not formed from the primary neurulation [23]. 

Based on this, it would be of great interest to know which levels of fetal hyperglycemia affect vertebral development, as well as which genes among those involved in this process are affected and what the day-to-day consequences of fetal hyperglycemia during primary and secondary neurulations are. All this would allow us to understand why there are so many differences between the pathological manifestations of CRS.

Since the patient in our study must have suffered continuous hyperglycemia during pregnancy, it is logical that this induced fetal hyperinsulinemia once the fetal pancreas began to function [25]. It is also logical to assume that the continuous and increasing hyperglycemia, produced by maternal supply, led to a continuous increase in insulin production by the fetus, further favored by the abundant fatty tissue that the fetus had, since fat cells require a large absorption of glucose, but are very resistant to the action of insulin [26].

The fact that the patient developed hypoglycemia a few hours after birth and had to be given a high dose of intravenous glucose for 4 days supports the idea of fetal hyperinsulinemia that was partially corrected sometime later. Similar findings have been previously reported [27,28]. 

Interestingly, although episodes of significant hypoglycemia did not occur again in the patient, his plasma glucose levels always remained below the normal range during the time he was treated at our Center, even though his diet was normal and, in addition, he was treated with GH, a counterregulatory hormone, since it produces a certain and transitory resistance to insulin [29]. Unfortunately, we did not analyze his plasma insulin values in any situation, something that would surely have given us more information about how the secretion of insulin was, although abdominal ultrasound studies indicated that his pancreas was normal. 

Another interesting aspect of this study was that plasma levels of IGF-I always remained below the normal range for their age, even despite receiving GH treatment. These data support the assumption of a slightly disproportionate insulin secretion, since although GH is the key factor for the hepatic production of IGF-I, for this to take place, a certain degree of glucose metabolism in the liver is needed [22]. Equally interesting is the fact that despite the permanently low levels of IGF-I, the patient began to grow normally, which was confirmed not only by the measurements carried out, but also by the values of plasma alkaline phosphatase, something apparently paradoxical [22]. 

After birth, the patient presented hypocalcemia, which, like hypoglycemia, had to be corrected with the intravenous administration of calcium for four days. A study carried out in 1983 indicated that fetuses of diabetic mothers had hypocalcemia due to parathyroid hormone (PTH) deficiency, and that these newborns also had hypocalcemia [30]. In this case, apart from neonatal hypocalcemia, none of the blood tests carried out during treatment at our Center showed plasma calcium values outside the normal range.

In this study, we followed the same methods as in our previous work on a patient with SRC CRS successfully rehabilitated [12], with the exception that BMRT therapy was now added to the rehabilitation guidelines. The reason for this was not only the young age of the patient, but also the fact that this type of therapy increases motor skills and motor control and integrates primitive reflexes. However, in this patient, as in others with this syndrome, no rehabilitation would have been successful if GH administration had not been used.

It has been known for years that the GH/IGF-I axis plays an extremely important role in neural development, but also in the treatment of neurological injuries [31]. The restorative effects of this hormone have already been widely described in very different types of brain injuries, by our group and several others, both in humans [32,33,34,35,36,37,38,39,40,41,42] and experimentally in other animal species [43,44,45,46,47,48,49,50,51,52].

In relation to the spinal cord, a series of studies indicate that there are cells with neurogenic potential in it. It is therefore feasible to think that GH may act on these cells in a similar way to how it does in the brain. In fact, the effects on neurogenesis that the hormone exerts at this level are even clearer than those it induces at the brain level, as indicated by studies carried out in transgenic mice expressing a GH antagonist [53]. The size of lumbar motoneurons in transgenic mice overexpressing GH was shown to increase in parallel to the increase in their body size, something that did not occur in their non-transgenic littermate controls [54]. Another significant finding indicates that the amount and enzymatic activity of acetylcholinesterase in the SC of GH-deficient rats is markedly reduced; since this enzyme is a marker of cholinergic neurons and their synapses, these data indicate that due to GH deficiency, the positive effects of the hormone on the proliferation of cholinergic synapses in the spinal cord of these animals do not exist [55]. 

Human studies indicate that there are also neurogenic activities in the SC of our species [56]. Nestin immunoreactive cells have been detected at the cervical, dorsal, and lumbar levels in subjects who died after a brain trauma, which indicates the existence of neural progenitors differentiating into neurons in the SC of these subjects [57]. This suggests that in the SC, there is a population of neural progenitor cells with the potential for proliferation, differentiation, and migration, as occurs in the brain. However, these characteristics seem to be able to occur up to a certain age, established around 18 years [58], which seems to be the determining factor for a favorable response to GH treatment in spinal cord injuries from a certain age, something that also coincides with our unpublished data in a series of spinal cord injuries treated with GH and rehabilitation. In any case, and given the very young age of our patient, it seems clear that, as in the other patient with CRS that we treated previously [12], the treatment with GH is, without a doubt, the factor responsible for his positive evolution.

As in our previous study [12], we cannot now state with certainty what the determining mechanisms were that led to the positive evolution of the treated patient. Only two possibilities come to mind, the same ones we previously described.

The first is that, as it does in the brain, the administration of GH induced an increase in the proliferation and differentiation of ependymal stem cells, leading to the development of new sensory and motor innervation (new spinal nerves arising from the last vertebra formed during somitogenesis), which would then be induced to act through rehabilitation, although the latter could also collaborate with GH to facilitate the differentiation (sensory and motor, afferent and efferent) of these new neural components. In this sense, it is very clear that rehabilitation alone does not play a significant role in the evolution of CRS.

Another possibility is that a network of new connections was formed from the last existing spinal nerve, and that rehabilitation has made them act on specific areas and in a specific way.

Regarding melatonin, it was administered to the patient to counteract the oxidative stress that he logically suffered due to his situation, although we did not measure it, as well as to protect the mitochondria and facilitate their activity [59].

In summary, for the second time in medicine, treatment with GH and rehabilitation enabled the patient to acquire a series of sensory and motor functions that he had not developed due to his CRS, including normal bladder and bowel function. It is foreseeable that the existing pelvis damage will also be able to be compensated soon by surgery and genetic engineering (artificial sacral bone for sacral agenesis), but in any case, the patient now leads a practically normal life. It is important to highlight that in the treatment of these cases, neither rehabilitation alone nor treatment with GH without rehabilitation would be effective. It is also important to point out that the earlier the age at which treatment begins, the sooner and better the positive results will be produced, as well as that from a certain age onwards, it does not seem feasible that this treatment could be effective.

## 4. Materials and Methods

### 4.1. Antecedents

The patient was a 2-month-old boy born at 33 weeks gestational age, who had been diagnosed (in utero ultrasonography) with caudal regression syndrome and sacral agenesis. According to the reports of his hospital of reference, the delivery was normal, with cephalic presentation and clear amniotic fluid. His mother was 37 years old, Caucasian, had had three previous normal pregnancies (from a previous marriage), and no abortions. However, since the age of 30, the mother had been suffering from type I diabetes mellitus treated with insulin, and her Hb A1c at delivery was 6.6% (higher than normal values). She smoked 6–7 cigarettes a day, but she did not have any other type of toxic habits. The patient was born from a non-consanguineal marriage. 

The child’s birth weight was 2800 g (p. 95, for his gestational age) and his length 44 cm (pp. 50–75). His Apgar score at birth was 9/10 (1 min/5 min), he was in good general condition, normal hydration, and normal perfusion. His skull and face were of a normal configuration and he had normotensive anterior fontanelle and no signs of respiratory distress. Cardiocirculatory studies showed rhythmic cardiac activity and present and symmetrical brachial and femoral pulses. His abdomen was soft and depressible, with no evidence of masses. His pupils were isochoric and normally reactive.

The molecular exams (Multiplex ligation probe amplification; MRC-Holland) carried out did not show any alteration in the subtelomeric regions analyzed; the karyotype was that of a normal male (46, XY) and the analysis of the genes that might have been involved in the appearance of the syndrome was also normal.

Osteoarticular studies at birth showed flattened buttocks, with shortening of the intergluteal groove and formation of dimples on the sides of the fissure. The feet were deformed (left clubfoot, non-reducible) and there was a contraction in the flexion of the knees and hips, which made it impossible to examine them. The femurs appeared to be hypoplastic and there were defects at the level of the tibia and fibula, more noticeable in the right lower limb. At the genital level, the impression was of a very small penis size, raft-shaped testes, and a narrowed anus.

Routine blood tests after birth: Hemoglobin 17.9 g/dL, Hematocrit 51.7%, Leukocytes 22.070, Platelets 550.000, Glucose 67 mg/dL, Creatinine 0.39 mg/dL, Urea 10 mg/dL, Total bilirubin 5.95 mg/dL, GPT 30 U/L, Sodium 133 mEq/L, Calcium 11.8 mg/dL, and CRP < 1.0 mg/L. Urine culture: Negative.

Interestingly, from the first hours of life, the patient presented hypoglycemia, despite enteral supplementation, so he required intravenous (IV) glucose at doses of up to 8 mg/kg/min. He also presented hypocalcemia, so he was administered IV calcium (2 mEq/kg). These glucose and calcium supplements were maintained until the 4th day of life, when blood glucose and calcium levels returned to normal values.

The orthopedic examination by the rehabilitation physician indicated that the patient had hips in non-reducible flexion of 90°, knees in extension with great limitation of flexion, being able to reach a maximum of 10° in the left knee, but nothing in the right. The left foot was in equinus varus and the right foot was cavo-supinated without associated equinus. There were no palpable quadricep contractions in either leg, no plantar flexion or dorsiflexion of the ankle in either foot, or flexion of the toes. There was an impression of overflowing urination and there was low anal tone and perianal dermatitis.

Neonatal jaundice was corrected two days later by phototherapy.

A new abdominal ultrasound showed that everything was normal except that the left kidney did not exist. The ultrasound of the hip showed that the iliac bones were fused with the pelvis and that both hips were dislocated without identifying the acetabulum, and a possible posterior location of the femoral heads on the iliac plates.

The magnetic resonance imaging (MRI) of the skull and spine indicated that there were no brain abnormalities, nor at the cervical and thoracic spinal cord level. However, the medullary cone was clearly deformed, ending abruptly at the level corresponding to the middle third of the T12 vertebral body. Its configuration was abnormal in the form of a club with the distal end absent, which was compatible with group I sacral agenesis. The spine, and consequently the spinal cord, ended at L4.

In summary, the patient was diagnosed in his hospital of reference with Renshaw type III–IV sacral agenesis with bilateral teratological dislocation of the hip, rigid hyperextended knees, and club feet. In addition, the left kidney had not been formed. 

To correct the hyperextension of the knees and the deformed feet, at two months of age, they started a treatment with serial casts, but hours after their placement, the patient began to present slow capillary refill and a slight drop in temperature in the midline, so the cast was removed. The parents were informed that their son would need several trauma surgeries and that he would never walk.

The parents then decided to come to our Medical Center to treat their son.

### 4.2. Admission to the Foltra Medical Center

**Figure 7:**
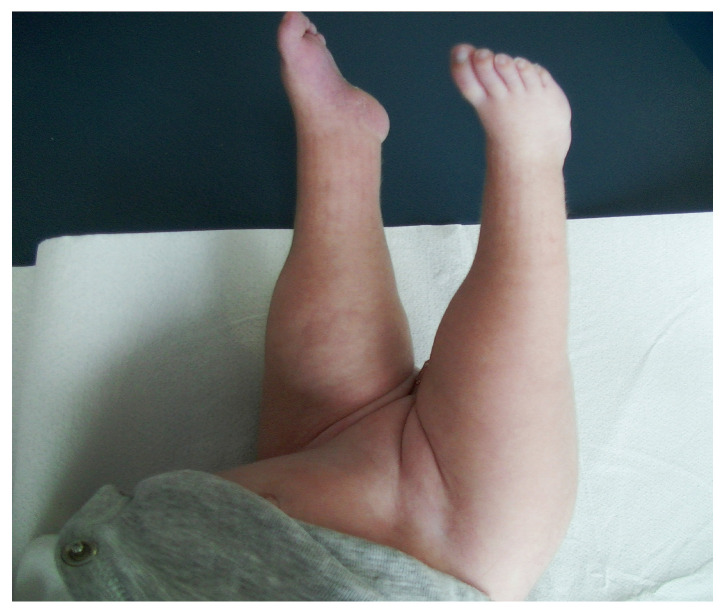
At admission. Observe the legs at an irreducible extension of practically 90°, the deformity of the legs, hypoplastic femurs, and the marked abundance of fat in the legs.

**Figure 8:**
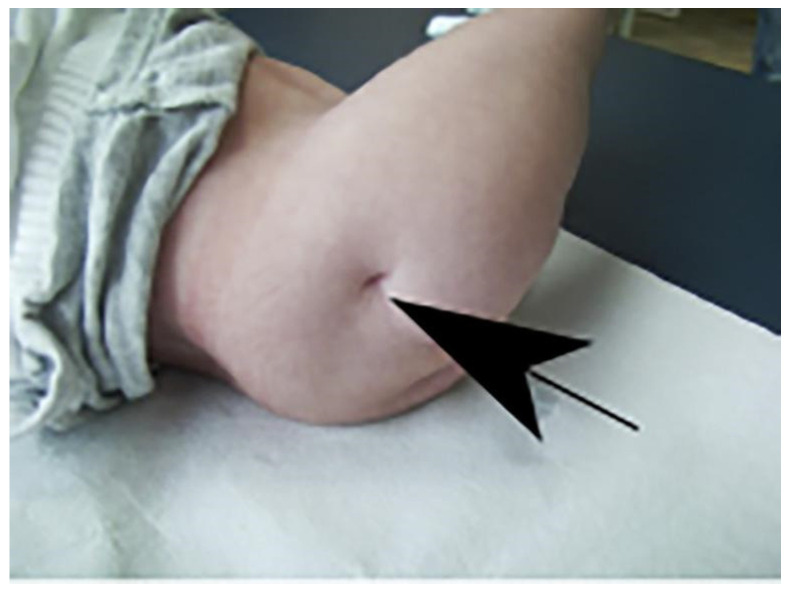
At admission. The image shows flattened buttocks with shortening of the intergluteal groove and formation of dimples on the sides of the fissure (black arrow).

The patient was admitted to our Center at two months and six days of age. The examination performed upon admission revealed sacral and left renal agenesis, a neurogenic bladder and intestine, and a lack of sensory and motor innervation of the lower limbs. In addition, his pelvis was flexed, his legs were in irreducible spastic extension of 90° without any movement, there were hypoplastic femurs, and his feet were deformed. He had a neurogenic bowel and bladder (continuous emission of feces and urine), hips in irreducible flexion of 90°, and an absence of motor activity in both legs, which were in irreducible extension of 90°. As described at birth, there were flattened buttocks, with shortening of the intergluteal groove and the formation of dimples on the sides of the fissure. The feet were deformed. The femurs appeared to be hypoplastic and there were defects at the level of the tibia and fibula, more noticeable in the right lower limb (Figure 7 and Figure 8), and there was apparent sensitivity up to L1–L2. 

The face was normally configured, although the head was disproportionately sized in relation to his age and the rest of the body.

The typical scales for evaluating child development milestones could not be performed due to repeated absences from therapies due to respiratory infections and bronchiolitis (in this case he had to remain in the hospital for a month) and the parents’ vacations. The only exception was the Gross Motor Function test (GMFM-88), carried out at admission and three months before discharge.

One month after admission, the patient underwent a CT-SCAN of the pelvis and legs, and the following month, an electroneurographic examination of the muscles of the limbs. In addition to these studies, during the episode of bronchiolitis that led to his hospitalization, he underwent an electroneurography of the members, a thoracoabdominal ultrasound, a radiological examination, and an MRI that in this case, analyzed the brain and spinal cord.

The treatment was like that previously published by our group [12]: the daily administration of Growth Hormone (GH) and melatonin, medical treatment, and physical rehabilitation. Although the treatment lasted only 18 months due to family problems, and of these 18 months, a total of about 4 months were interrupted by the parents’ vacations and 3 respiratory infections of the patient, the evolution upon discharge was very positive; the legs had grown and moved in all directions, the sensitivity in the legs was complete, the neurogenic bladder and intestine were no longer present, he was able to sit up and roll over on his own, and ten months after discharge, the patient was able to walk with the aid of a back walker and move independently on an electric motorcycle. The medical treatment did not produce any adverse side effects.

Based on the very positive results achieved in another patient with this syndrome [13], a medical treatment was prescribed consisting of the administration of Growth Hormone (GH, Genotonorm, Pfizer, Spain, 0.04 mg/kg/day, 5 days a week for three months followed by 15 days of rest), liquid melatonin (20 mg/day, orally, without interruption, at night before going to sleep), and physical rehabilitation consisted of daily sessions of Physiotherapy, hydrotherapy (working in water with a physiotherapist), and BMRT (Blomberg Rhythmic Movement Training), all of them in 45-min sessions, 5 days a week. 

Upon admission, a general blood test was performed, and it was repeated every three months. This blood analysis (hematimetry and biochemistry) included plasma values of TSH, fT4, morning cortisol, and IGF-I.

**Figure 9:**
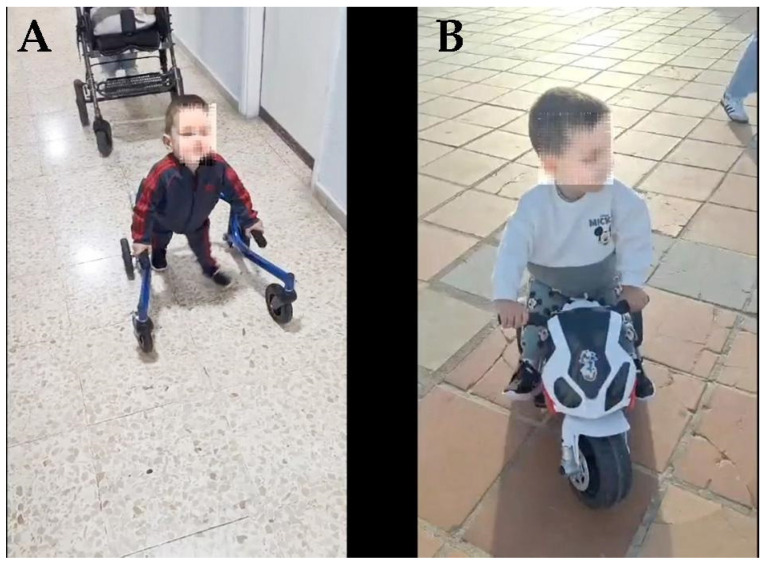
10 months after discharge. (**A**): Walking with the support of a back walker. Note the different position of the legs for walking. (**B**): Riding a small electric motorcycle.

Eighteen months after his admission to our Center, due to family problems, the patient returned to his hometown with his family. Before leaving our Center, the family was given instructions on how to continue the therapies at home, 1000 km away from our Center, but we do not know how they carried them out, even though 10 months later the family sent us some videos showing how the patient walked with the support of a back walker and riding a small electric motorcycle (Figure 9A,B; Movie S1).

Studies and treatments were conducted according to our protocols and in compliance with the Spanish legislation for using GH “off label” and the Code of Ethics of the World Medical Association (Declaration of Helsinki). Signed informed consent for using GH and melatonin was obtained from the legal representatives of the patient.

The photographs and videos were taken by therapists. Videos after discharge were taken and sent by the parents of the patient. The parents of the patient gave signed informed consent for the publication of the images and videotapes of the patient.

## 5. Conclusions

GH treatment and specific rehabilitation are essential for the acquisition of sensory and motor innervation in patients with CRS. This treatment should be carried out at the earliest possible age to induce the proliferation and differentiation of stem cells in the medullary ependyma before they decrease or disappear.

## Data Availability

The original contributions presented in this study are included in the article/Supplementary Material. Further inquiries can be directed to the corresponding author.

## Supplementary Materials

The following supporting information can be downloaded at: https://www.mdpi.com/article/10.3390/ijms26041627/s1.
