# Supplementary figures and images for: Positive Evolution of a Child Suffering from Caudal Regression Syndrome and Agenesia Sacra After Treatment with Growth Hormone and Rehabilitation
Source: Int J Mol Sci. 2025 Feb 14;26(4):1627. doi: 10.3390/ijms26041627 (PMC11855933; doi:10.3390/ijms26041627)

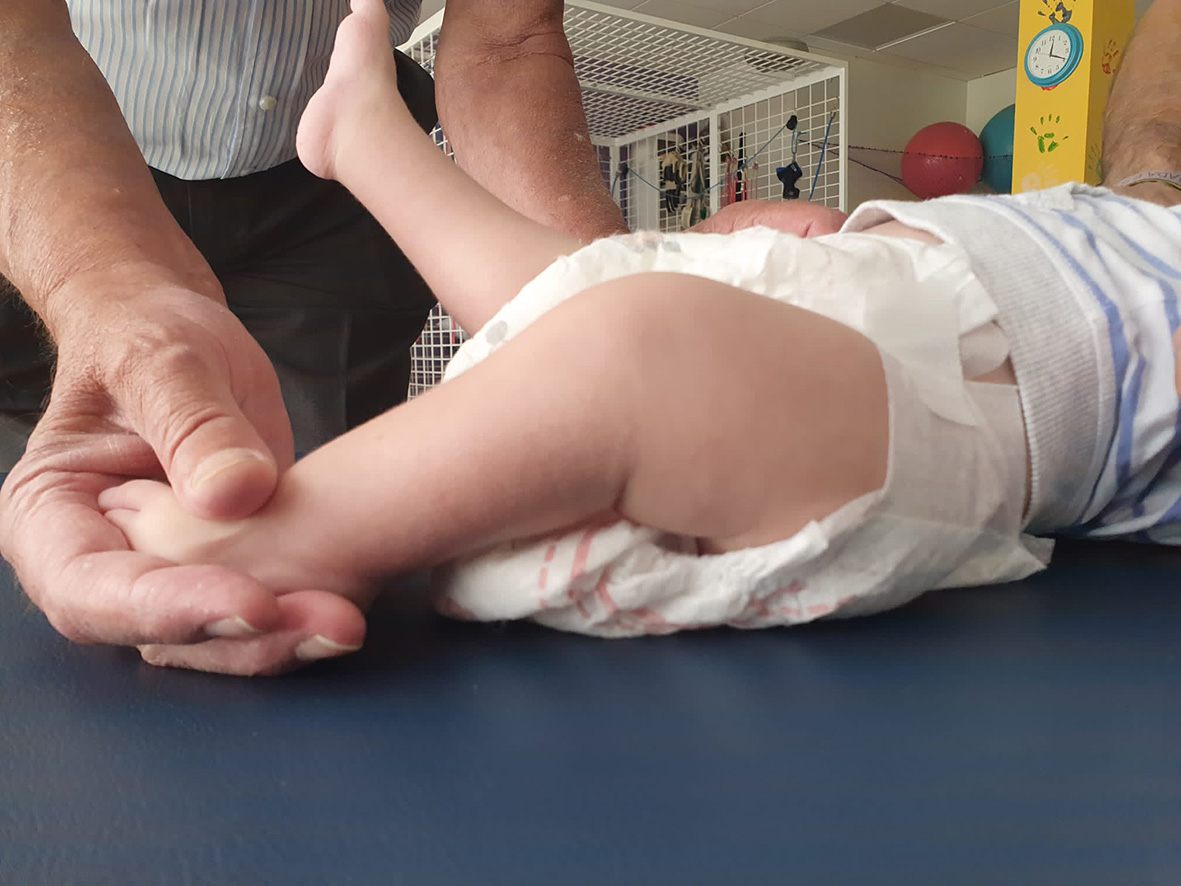

Supplement: Supplementary file 1 [file ijms-26-01627-s001.zip › Figure S1.tiff]

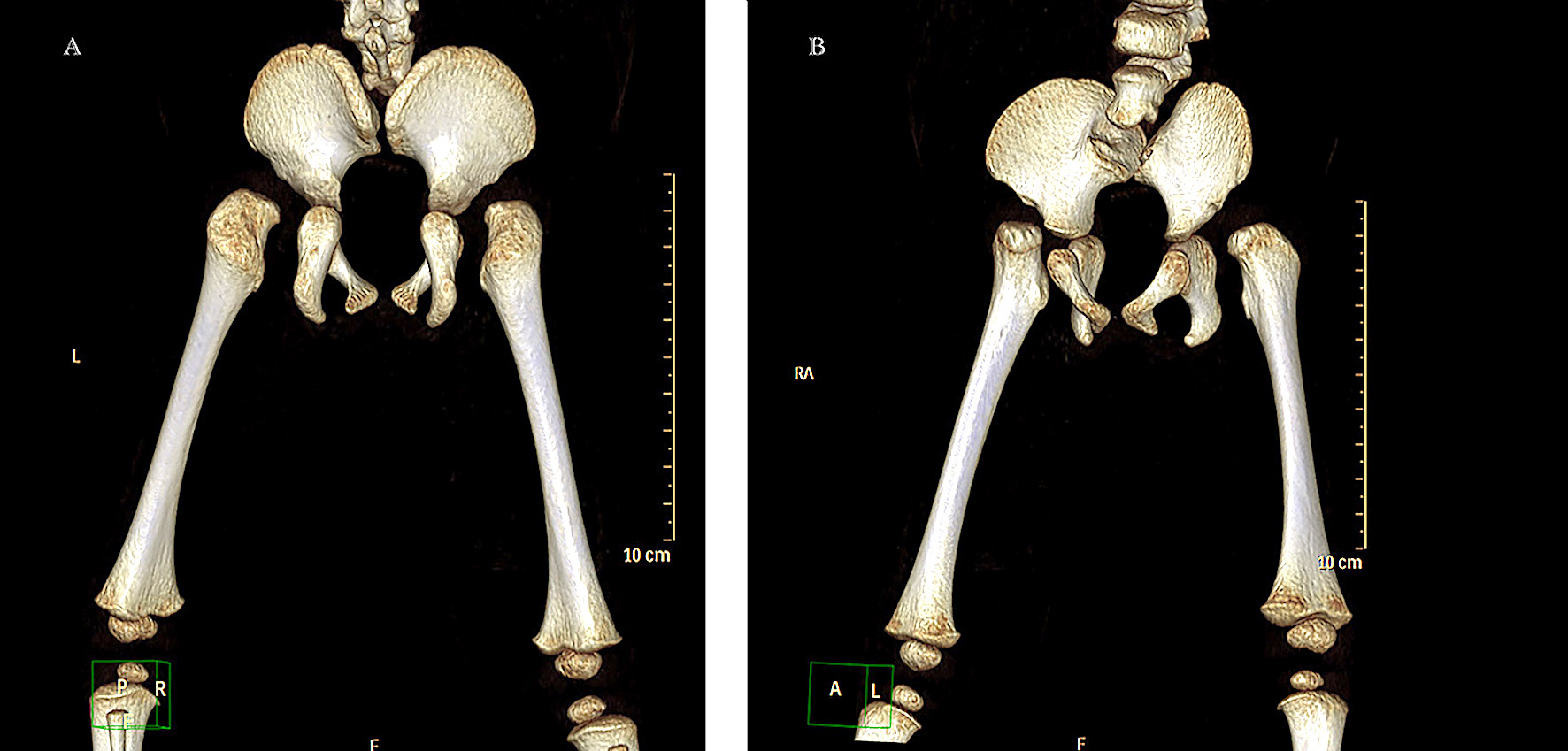

Supplement: Supplementary file 1 [file ijms-26-01627-s001.zip › Figure S2 A and B.tiff]

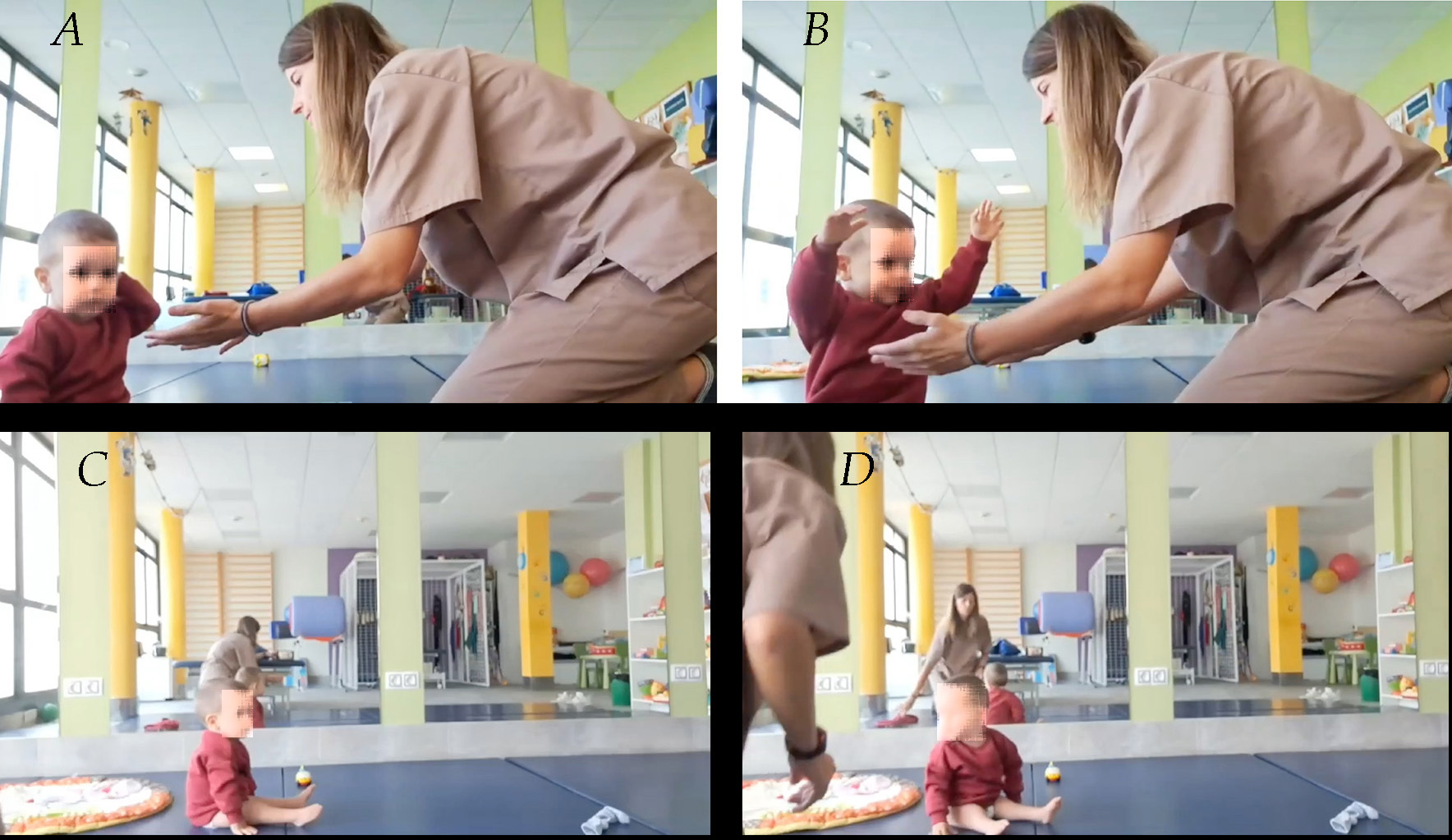

Supplement: Supplementary file 1 [file ijms-26-01627-s001.zip › Figure S3.tiff]

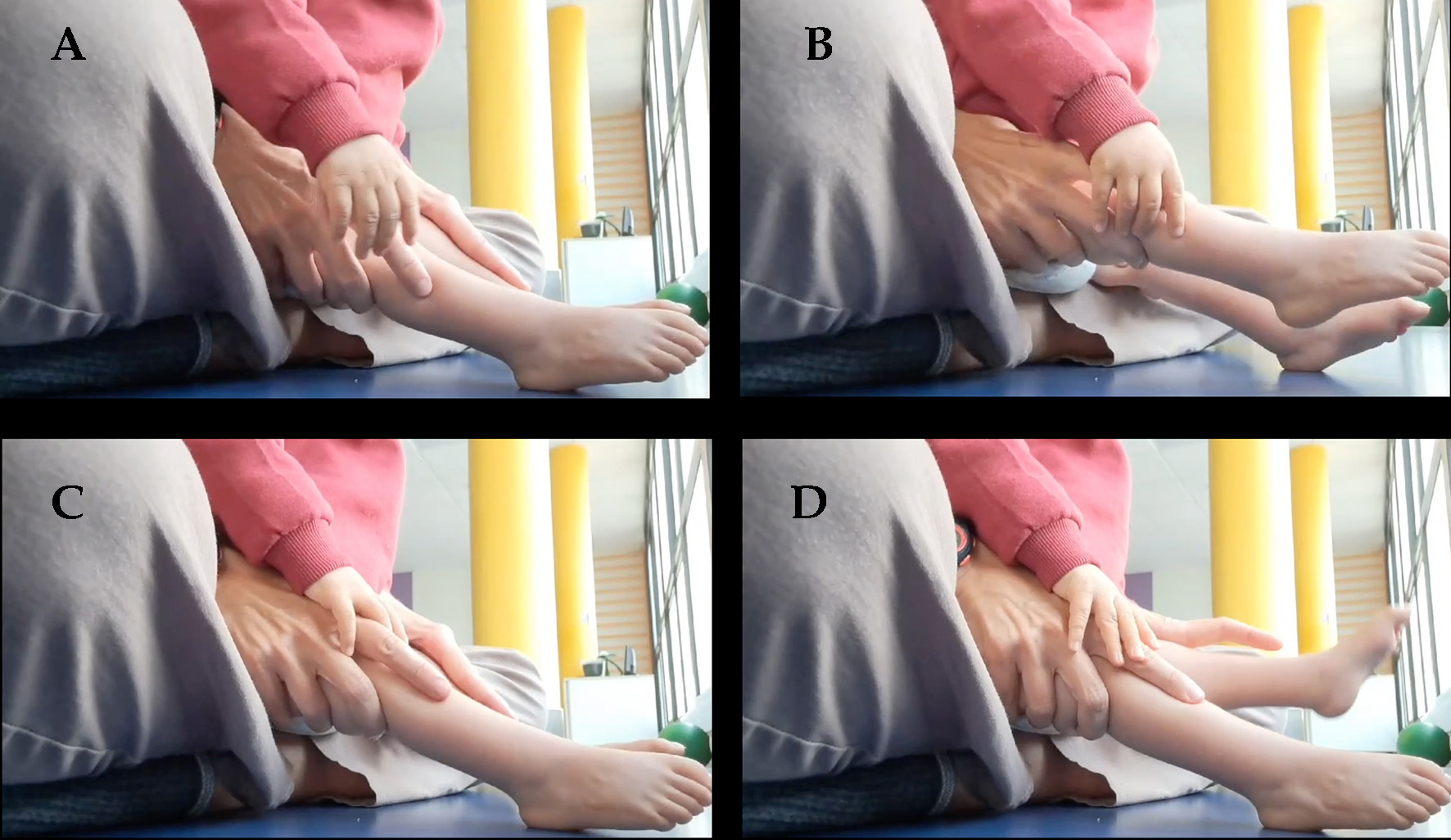

Supplement: Supplementary file 1 [file ijms-26-01627-s001.zip › Figure S4.tiff]

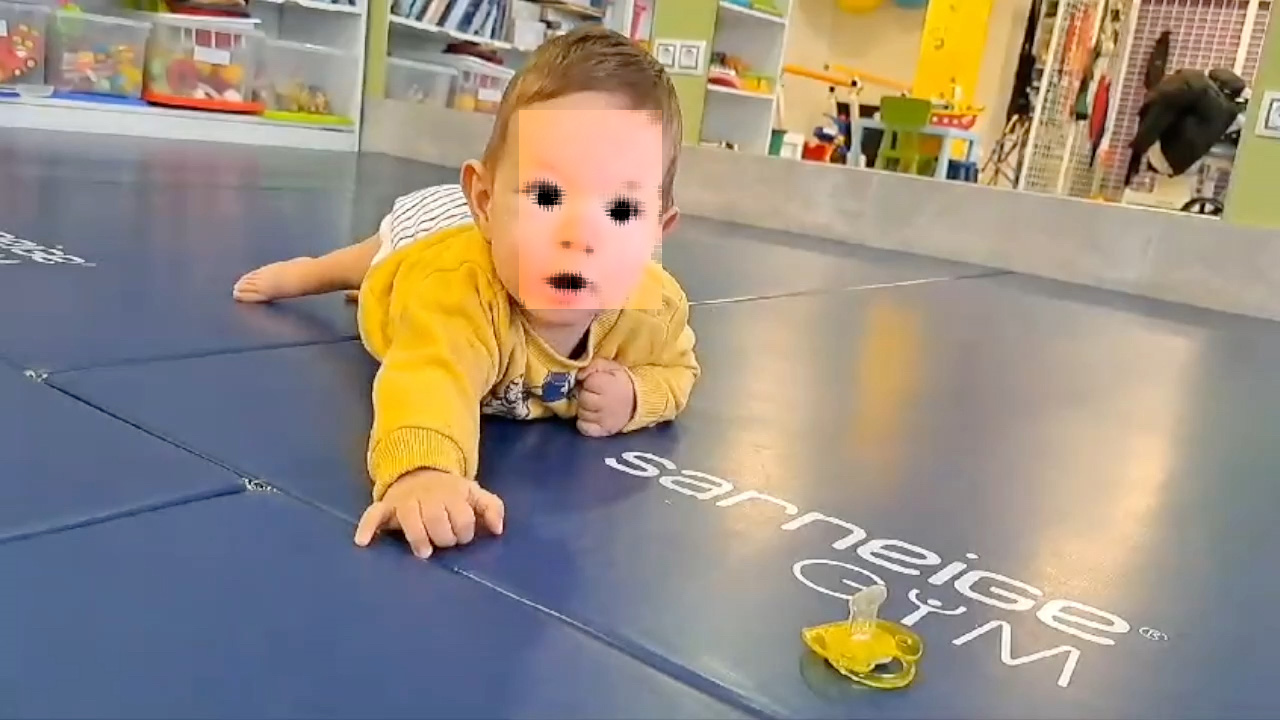

Supplement: Supplementary file 1 [file ijms-26-01627-s001.zip › Figure S5 Gall.tiff]

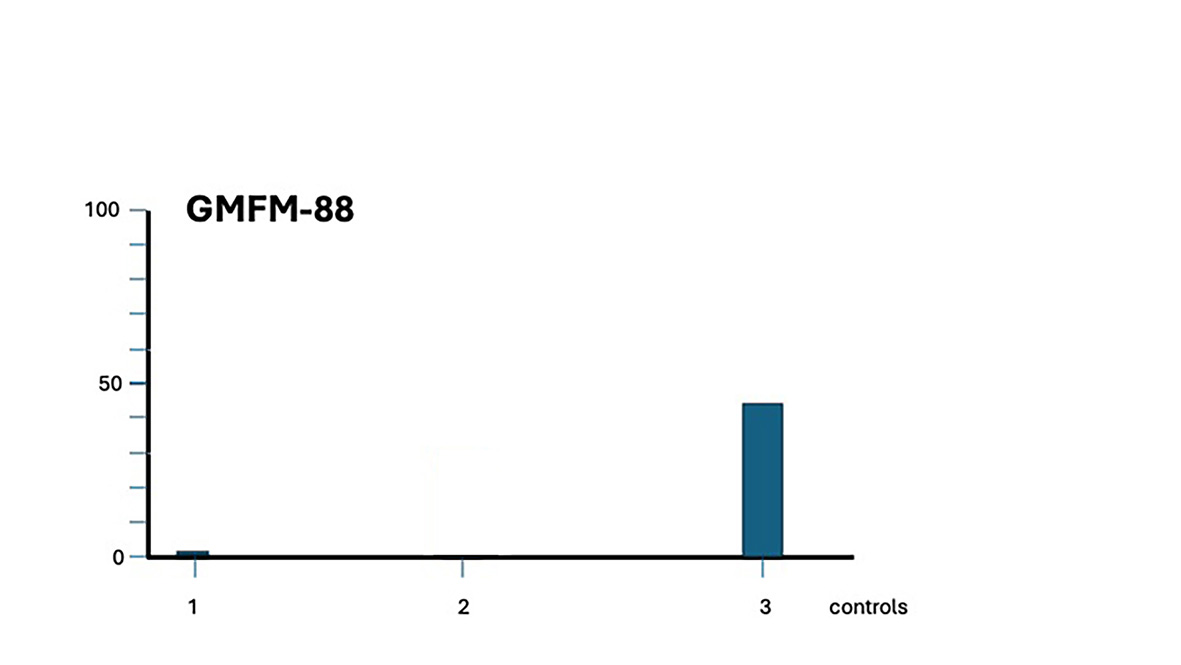

Supplement: Supplementary file 1 [file ijms-26-01627-s001.zip › Figure S6 .tiff]

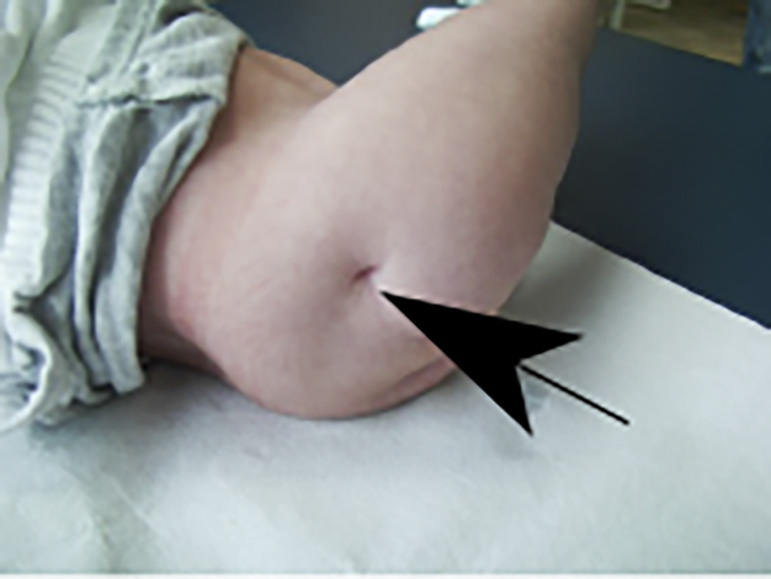

Supplement: Supplementary file 1 [file ijms-26-01627-s001.zip › Figure S8.tiff]

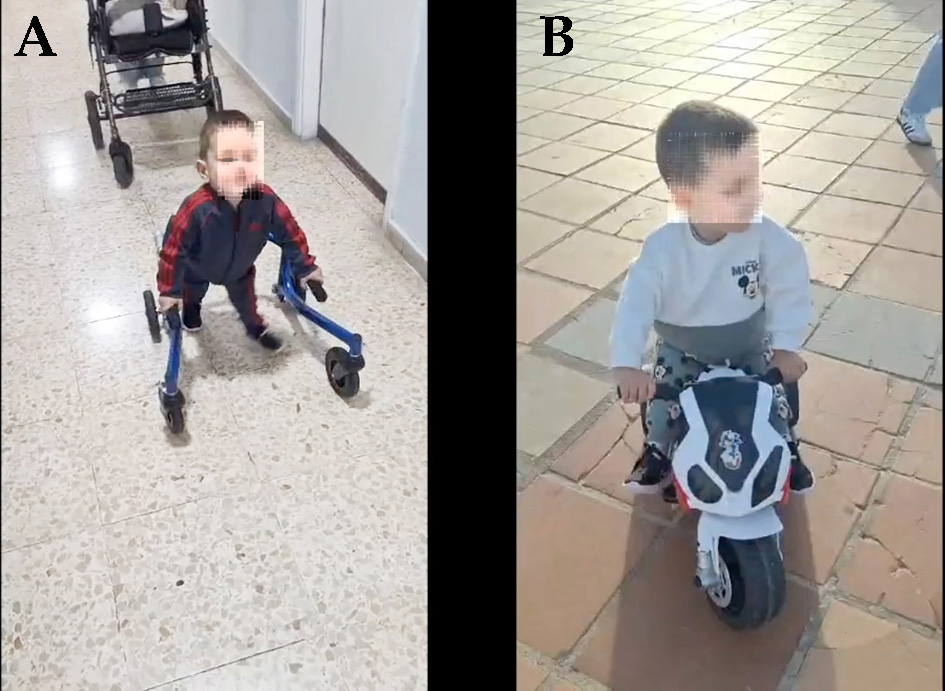

Supplement: Supplementary file 1 [file ijms-26-01627-s001.zip › Figure S9.tiff]
